# Dissecting fruit weight and quality traits in Australian passion fruit through genetic linkage mapping and QTL analysis

**DOI:** 10.3389/fpls.2026.1755188

**Published:** 2026-04-02

**Authors:** Xinhang Sun, Peter Bundock, Vivi Arief, Bruce Topp, Mobashwer Alam

**Affiliations:** 1 Centre for Horticultural Science, Queensland Alliance for Agriculture and Food Innovation, The University of Queensland, Nambour, QLD, Australia; 2 Southern Cross Plant Science, Southern Cross University, Lismore, NSW, Australia; 3 School of Agriculture and Food Sustainability, The University of Queensland, St Lucia, QLD, Australia

**Keywords:** breeding, genetic map, linkage analysis, *Passiflora edulis*, passion fruit, QTLs

## Abstract

**Introduction:**

Passion fruit (*Passiflora edulis*) is a commercially significant horticultural crop in tropical and subtropical regions. However, the development of high-yielding cultivars with superior fruit quality is currently hindered by limited understanding of the genetic architecture of complex fruit traits. This study aimed to construct a high-density genetic linkage map and elucidate the genomic regions underlying fruit weight and key quality traits, including total soluble solids and pH.

**Methods:**

A bi-parental mapping population was developed from a cross between the Australian elite cultivars ‘Tom’s Special’ and ‘Lacey’ and high-throughput genotyping was performed on the parents and 184 full-sib progeny to identify single-nucleotide polymorphisms (SNPs).

**Results and discussion:**

The genomic best linear unbiased prediction (GBLUP) analysis indicated low genomic heritability estimates ranging from 16.7% to 38.1%. Significant positive correlations were detected between fruit weight and total soluble solids for both observed and predicted values. The linkage map constructed with the Cross-Pollination (CP) model comprised 8223 SNP markers distributed across 9 chromosomes, spanning a total length of 706.35 cM. Using the interval mapping algorithm, five quantitative trait loci (QTLs) were identified based on observed phenotypic data. These QTLs were located on three linkage groups, accounting for 11.51% to 19.40% of the phenotypic variation, with the highest LOD score of 5.48. Functional gene annotations of significant QTLs revealed *bHLH68*, *NHX1*, *AGP4*, and *IAA18* as putative candidates, suggesting carbohydrate allocation, vacuolar pH regulation, cell wall dynamics, and Auxin-mediated growth as potential mechanisms underlying the observed trait variation. Collectively, these findings provide a robust foundation for future fine-mapping and the implementation of marker-assisted selection to accelerate genetic gain in passion fruit.

## 1 Introduction

Passion fruit (*Passiflora edulis*) is a highly valued crop in tropical and subtropical regions with significant economic potential for fresh consumption, processed products, and ornamental use (Santos et al., 2024). Global demand for passion fruit has increased substantially due to its rich nutritional composition, including essential minerals, dietary fiber, vitamins, and various bioactive compounds, as well as remarkable medicinal properties such as antioxidant, anti-inflammatory, and neuroprotective effects (Xia et al., 2021; Fang et al., 2023; Pereira et al., 2023; Zhang et al., 2023). Brazil is the world’s largest producer and consumer of passion fruit, followed by Colombia and Indonesia (Weyya et al., 2024). In Australia, passion fruit is cultivated across approximately 280 hectares, with an annual production of 4,711 tons in 2022-2023 (Sun et al., 2025b). Despite its potential, the Australian passion fruit industry is constrained by a lack of improved cultivars to meet the demands of both producers and consumers (Sun et al., 2025a). Domestic production is heavily reliant on the purple cultivars, ‘Sweetheart’ and ‘Misty Gem’, and the Panama cultivar ‘Pandora’. This narrow genetic base of current cultivars increases their vulnerability to both abiotic and biotic stresses, resulting in yield losses and decreased fruit quality (Asande et al., 2023). Therefore, the development of novel cultivars using efficient breeding strategies is imperative to ensure long-term sustainability and profitability of the industry.

Conventional breeding faces challenges such as high costs, long breeding cycles, and a lengthy juvenile phase (Longhi et al., 2013). In contrast, marker-assisted breeding offers a more efficient alternative, enabling the selection of economically significant traits at the seedling stage, thereby accelerating the breeding process (Varshney et al., 2005). Passion fruit breeding has traditionally focused on phenotypic selection, relying on visual assessments of performance (da Silva Pereira et al., 2017). Despite the growing application of marker-assisted selection, its use in passion fruit breeding remains limited (Cerqueira-Silva et al., 2014; Viana et al., 2017). Breeding for enhanced yield and fruit quality in passion fruits is complex due to polygenic nature of these traits (Chavarría-Perez et al., 2020). Understanding the genetic architecture underlying these quantitative traits is therefore a critical bottleneck in passion fruit improvement (Maan et al., 2023). The integration of genetic linkage mapping and quantitative trait locus (QTL) analysis has proven effective for dissecting complex traits across a wide range of crop species (Hasan et al., 2021). For example, QTL mapping has been constructed for growth vigor in kiwifruit (Li et al., 2025), berry shape in grape (Wu et al., 2023) and fruit quality traits in guava (Maan et al., 2023). In *Passiflora*, QTL studies have been reported for fruit characteristics in *P. alata* (da Silva Pereira et al., 2017), stem rot resistance in *P. edulis* (Wu et al., 2025), and resistance to cowpea aphid-borne mosaic virus (CABMV) in a segregating population of *Passiflora* (Santos et al., 2019). However, there is still a lack of information regarding QTLs underlying fruit weight and quality traits in *P. edulis*.

Previous studies have utilized different molecular marker types for the genetic mapping in *Passiflora* such as amplified fragment length polymorphisms (AFLPs) combined with microsatellite markers (Oliveira et al., 2008), random amplified polymorphic DNA (RAPD) markers (Carneiro et al., 2002) as well as inter-simple sequence repeat (ISSR) and simple sequence repeat (SSR) (Santos et al., 2019). While these studies provided foundational insights, their limited marker density constrained map resolution and the precision of QTL detection. Whole-genome profiling utilizing single nucleotide polymorphism (SNP) markers has demonstrated considerable potential for generating high-density genetic maps that can further enhance the identification of markers tightly linked to QTLs (Wu et al., 2025). Diversity Arrays Technology sequencing (DArTseq™) provides a cost-effective and high-throughput approach for genome-wide genotyping without any prior sequence information (Schouten et al., 2012). This platform offers distinct advantages over standard Genotyping-by-Sequencing (GBS) and whole-genome resequencing, particularly for species with limited genomic resources like *Passiflora*. DArTseq utilizes methylation-sensitive restriction enzymes (e.g., *PstI*) to achieve genome complexity reduction. This approach preferentially targets gene-rich and subtelomeric regions, thereby enhancing the functional relevance of mapped markers (Wenzl et al., 2004). The utility of DArT markers in constructing genetic linkage maps has been well-established across various plant species, including sorghum (Mace et al., 2008), apple (Schouten et al., 2012), and strawberry (Sánchez-Sevilla et al., 2015).

Compared to previous QTL research emphasizing disease resistance or other *Passiflora* species, this study focuses on elucidating the genetic basis of commercially important traits relevant to fresh and processed markets in *P. edulis*. Accordingly, the objectives of this study were to: 1) evaluate the genetic variability within an Australian bi-parental *P. edulis* population; 2) construct a high-density genetic linkage map based on the genome-wide DArTseq-derived SNP markers; 3) identify QTLs associated with fruit weight and quality traits.

## 2 Materials and methods

### 2.1 Plant materials

An F1 population of 184 full-sib progeny, derived from a cross between two highly heterozygous elite cultivars ‘Tom’s Special’ and ‘Lacey’ under the Australian National Passionfruit Breeding Program (PF19000), was phenotyped to evaluate fruit-trait segregation and genotyped to facilitate the identification of marker-trait associations. The ‘Tom’s Special’ cultivar has vigorous vines that produce large, round to oval fruits. It exhibits high internal quality, characterized by sweet, juicy orange pulp and a high pulp recovery of approximately 49%.The average fruit weight is around 90 g (Rigden, 2011). The ‘Lacey’ cultivar is distinguished by its resistance to key diseases, especially fusarium wilt (*Fusarium oxysporum* f. sp. *passiflorae*). It produces medium-sized, purple-skinned fruit with an average weight of 70 g, typically round to slightly oval in shape, and a firm rind that supports marketability (Rigden, 2011).

### 2.2 Mapping population development

The mapping population was developed through controlled hybridization between the female parent ‘Tom’s Special’ and the male parent ‘Lacey’. Flower buds of ‘Tom’s Special’ were selected one day prior to anthesis and emasculated at the bud stage by carefully removing anthers with sterilized forceps to prevent self-pollination. Emasculated flowers were immediately bagged with glassine paper bags to exclude insects and avoid unintended pollen contamination. The following day, hand pollination was conducted using freshly collected pollen from fully opened flowers of ‘Lacey’. Pollen was manually transferred to the receptive stigmas of emasculated ‘Tom’s Special’ flowers using a paint brush. Pollinated flowers were re-bagged and labelled to ensure accurate cross identification. After approximately 8 weeks, seeds were extracted from fully ripened fruits and germinated in seedling trays containing a 1:1 mixture of soil and commercial potting substrate in a glasshouse. Ninety days after, a total of 184 seedlings were transplanted to the field trial located in Burringbar NSW (28°26’49.4”S 153°25’30.4”E), at a spacing of 3 m between rows and 2 m between plants. The plants were managed under drip irrigation with regular weed control, fertilizer application and other agronomic practices according to regional recommendations (Rigden, 2011).

### 2.3 DNA extraction and DArTseq genotyping

Young leaf tissue was collected from both parental plants and 184 progeny individuals, placed into 15 mL Falcon tubes. The samples were rapidly frozen in liquid nitrogen and stored at -80 °C until further processing. Genomic DNA was extracted from approximately 500 mg of leaf tissue using the cetyltrimethylammonium bromide (CTAB) method (Doyle et al., 1991). DNA integrity was assessed via electrophoresis on a 0.8% agarose gel, while its concentration was quantified using a spectrophotometer (Pereira et al., 2015). The DNA samples were subsequently standardized to a concentration of 50 ng/μL (Oluoch et al., 2018). The extracted DNA was then submitted to Diversity Arrays Technology Pty Ltd. (Canberra, Australia) for DArTseq™ genotyping. Genome complexity reduction was performed utilizing a combination of restriction enzymes optimized for *Passiflora* species, following the protocol described by Curtolo et al. (2017). Genomic DNA libraries were prepared according to the DArTseq™ protocol and sequencing was performed using the HiSeq 2000 platform (Illumina, USA). Raw sequencing data were processed using DArTsoft14 analytical program integrated with the DArTdb laboratory information management system to identify SNP markers. Marker quality filtering was conducted using the VCFtools software (Danecek et al., 2011), and only markers with a call rate ≥80% (--max-missing 0.8) and a minor allele frequency (MAF) ≥0.05 (--maf 0.05) were retained for downstream analyses.

### 2.4 Parentage analysis

Parent-progeny relationship using the *apparent* package (Melo and Hale, 2019) was evaluated to confirm that all progeny originated from the intended cross rather than self-fertilization. Expected progeny (EPij) genotypes, representing the hypothetical offspring of parents i and j, were inferred from homozygous markers between two parents. Pairwise genetic distances between each EPij and all potential offspring k (POk) were then calculated using the Gower Dissimilarity (GD) metric which provides evidence that i and j are the true parents of offspring k. Only triads (parent pairs + progeny) including the original maternal parent were considered. Significant triads were identified by applying a Dixon test to the distribution of all GD (EPij|POk) values (Melo and Hale, 2019), which detects a gap separating likely true parent-offspring triads from spurious associations. Selfed progeny were detected as individuals exhibiting all homozygous loci present in the maternal parent (Michael et al., 2023).

### 2.5 Fruit trait evaluation

Between June and August 2017, an average of six fruits per plant were collected weekly to examine the following fruit traits: fruit weight (g), sugar concentration (°Brix), and pH content. Fruit weight was measured using a digital scale (Mettler-Toledo Ltd., Australia). Brix content was quantified using a handheld digital pocket refractometer with a scale ranging from 0 to 53% (ATAGO Co., Ltd., Japan), and pH was determined using a calibrated pH meter (Mettler-Toledo Ltd., Australia).

### 2.6 Statistical analysis

The linear mixed effects model for genomic best linear unbiased prediction (GBLUP) was used to estimate genotypic variation as well as heritability for each trait in ASReml-R (Butler et al., 2017). The model is expressed as:


y=Xβ+Zγ+e

where y is a vector of phenotypic values; β is a vector of fixed effects including the mean; γ is a vector of random genetic effects; e is a vector of residuals; *X* and *Z* are the corresponding design matrices for β and γ, respectively. The random genetic effects (γ) were modelled using the genomic relationship matrix (G_m_), derived from SNP marker information according to the method described by VanRaden (2008). The proportion of the additive genetic variance captured by SNPs to the total variance, known as genomic heritability (*h^2^*) was calculated as:


$$h^{2}=\frac{\sigma_{a}^{2}}{\sigma_{a}^{2}+\sigma_{e}^{2}}$$

where *σ_a_^2^* is the additive genetic variance captured by SNP markers and *σ_e_^2^* is the residual variance (Zhao et al., 2021). Pairwise Pearson’s correlation coefficients were calculated among fruit traits using both the predicted values from GBLUP and the observed phenotypic data for the progeny (da Silva Pereira et al., 2017).

### 2.7 Genetic map construction and QTL analysis

A genetic linkage map was developed with the CP (Cross-Pollination) model for the outbreeding species in Lep-MAP3 software (Rastas, 2017). Prior to analysis, markers exhibiting >10% missing data, low minor allele frequency<0.05, or identified as non-informative or segregation distortion (dataTolerance = 0.001) by *Filtering2* module were removed. The remaining high-quality markers were grouped into distinct linkage groups using the *SeparateChromosomes2* module with a minimum logarithm of odds (LOD) threshold of 3.0 (Maan et al., 2023). Marker ordering within each linkage group was performed using the *OrderMarkers2* module based on Kosambi’s mapping function with sex-averaged genetic distances expressed in centiMorgans (cM). The linkage map was visualized with the LinkageMapView package (Ouellette et al., 2018).

QTL mapping was conducted using interval mapping (IM) in R/qtl software (Broman et al., 2003). The 4-way cross model in R/qtl was selected because the analyzed population derived from a cross between two outbred heterozygous parents (He et al., 2011). Genome-wide scans with a single-QTL model were performed using the scanone() function. The genome-wide significance threshold (α = 0.05) was determined using 1,000 permutations. Because permutation-based thresholds can be overly conservative, loci exceeding a fixed LOD score of 3.0 were also reported as suggestive QTLs (Lander and Kruglyak, 1995). QTL peaks were defined by the highest LOD scores, with confidence intervals estimated using the 1.5-LOD drop method. Significant loci were incorporated into a multiple-QTL model constructed using makeqtl() and fitted with Haley-Knott regression via fitqtl(), which was used to estimate the percentage of phenotypic variance explained (PVE%) by each QTL. QTLs with a PVE greater than 10% and a LOD score exceeding 3.0 were classified as major QTLs, whereas those below these thresholds were considered minor QTLs (Maan et al., 2023). The additive and dominance effects of QTLs were estimated according to the method described by Falconer and Mackay (1996). To evaluate the allelic effects of the identified QTLs, the phenotypic distributions across genotype classes were visualized using box plots generated with the ggplot2 package (Wickham, 2016), based on alleles at the peak marker. Differences among genotypic classes were assessed using Welch’s ANOVA, followed by pairwise t-tests with Bonferroni correction.

### 2.8 Candidate gene prediction

The nucleotide sequences of peak markers were aligned to the annotated Australian *P. edulis* reference genome (unpublished data) using BLASTN (Altschul et al., 1990) to obtain their physical positions. The genes located within a ±100 kb physical candidate window of these markers were identified using BEDTools (Quinlan and Hall, 2010) via the Galaxy platform (Afgan et al., 2018). For candidate genes lacking functional annotations in the reference genome, putative functions were inferred through BLASTP searches of their predicted protein sequences against NCBI non-redundant protein database (Altschul et al., 1997).

## 3 Results

### 3.1 Parent-progeny relationships and marker quality

Parentage analysis was conducted to identify the progeny resulting from self-pollination. Six individuals were classified as selfed from significant triads (*P* < 0.001), with GD (EPij|POk) values ranging from 0.016 to 0.048 (**Supplementary Table 1**). These selfed individuals were subsequently excluded from the progeny.

In addition, a total of 11,506 SNPs were selected based on >80% call rate and >0.05 MAF for further phenotypic and genotypic analyses. These markers were found to be highly informative, with an average polymorphic information content (PIC) value of 0.42 (**Supplementary Figure 1**). Approximately 59% of markers (6,833 SNPs) were classified within the highest PIC range (0.4 to 0.5).

### 3.2 Phenotypic and genotypic data analysis

Our results suggest low genomic heritability for all three fruit traits from 17% to 38% (**Table 1**). Fruit weight had the highest genomic heritability (38.1%) followed by Brix (30.7%), while pH had the lowest genomic heritability (16.7%).

**Table 1:** Estimate of the additive genetic (*σ_a_^2^*), phenotypic (*σ_f_^2^*), residual variances (*σ_e_^2^*) and genomic heritability (*h^2^*) for three fruit traits assessed from GBLUPs.

Traits	*σ_a_^2^*	*σ_f_^2^*	*σ_e_^2^*	*h^2^*
BR	0.997	3.246	2.249	0.307
PH	0.005	0.031	0.026	0.167
FW	164.916	432.681	267.765	0.381

**Figure 1:**
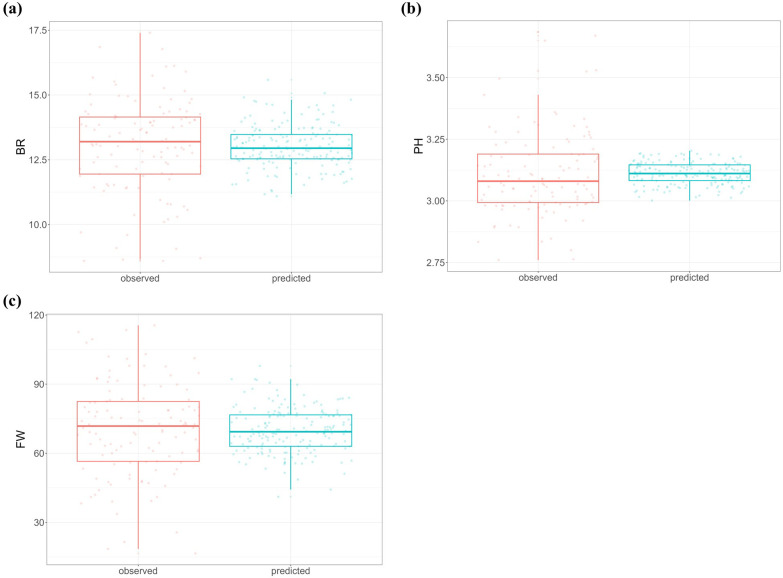
Observed phenotypic values and predicted values (GBLUPs) for three fruit traits: **(a)** Brix (BR), **(b)** pH (PH), and **(c)** fruit weight (FW).

Only 121 progeny seedlings from 184 produced a sufficient number of fruits for trait evaluation. Less variation was observed for the predicted values than the observed phenotypes for all three traits (**Figure 1**). Compared to pH, there was greater variation in the predicted values for Brix and fruit weight traits (**Figures 1a**, **c**).

### 3.3 Correlations between traits

**Figure 2:**
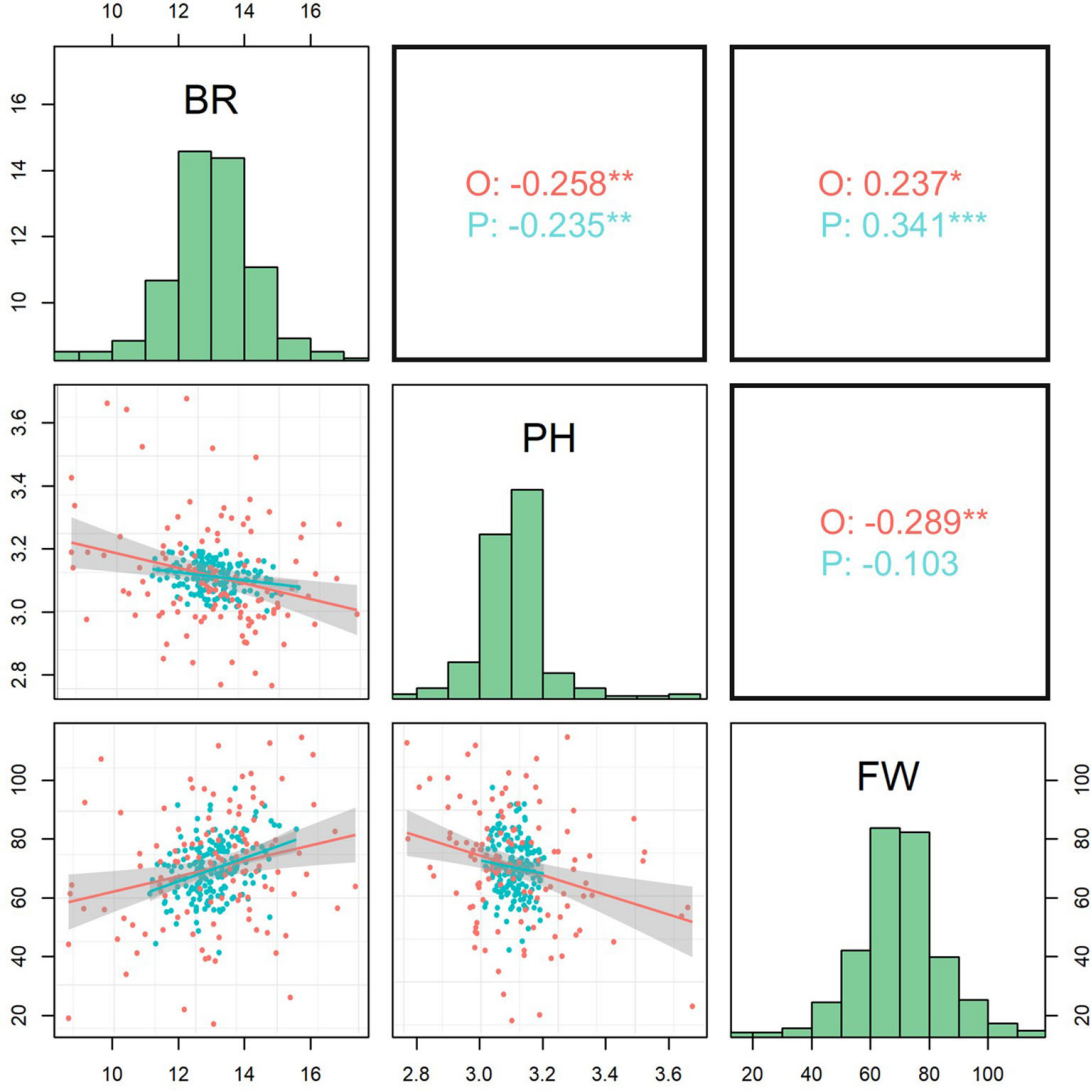
Scatter plots (below diagonal), Pearson correlations (above diagonal), with significance levels indicated as: *p < 0.05, **p < 0.01, ***p < 0.001, and histograms (diagonal) based on observed (O; red colour) and predicted (P; blue colour) values of three traits. BR, Brix; PH, pH content; FW, Fruit weight.

Pearson’s pairwise correlation analysis was conducted for both observed phenotypic data (r_o_) and predicted values (r_p_) to assess the strength and direction of the relationships among the three fruit traits (**Figure 2**). Significant positive correlations were observed between Brix and Fruit weight for both the observed (r_o_ = 0.237; *P* = 0.010) and predicted values (r_p_ = 0.341; *P* < 0.001). The regression analysis also indicates that fruit weight increased with higher Brix levels. In contrast, there were significant negative correlations between Brix and pH for both the observed (r_o_ = -0.258; *P* = 0.005) and predicted values (r_p_ = -0.235; *P* = 0.002). Furthermore, pH was significantly negatively correlated with fruit weight (r_o_ = -0.289; *P* = 0.002) based on the observed phenotypic data. In addition, three fruit traits, especially Brix and fruit weight, appear to be normally distributed based on the combined observed and predicted data (**Figure 2**).

### 3.4 Linkage map

A genetic linkage map comprising 9 linkage groups (LGs) was constructed using 8,223 polymorphic markers. The sex-averaged map spanned a total length of 706.35 centimorgans (cM), with an average inter-marker distance of 0.09 cM (**Table 2**). Among the linkage groups, LG1 was the longest at 111.54 cM, while LG9 was the shortest at 50.46 cM. LG1 also had the highest number of SNPs (1512), whereas LG9 had the fewest (494). The average length of the linkage groups was 78.48 cM and the average genetic distance between markers ranged from 0.07 (LG1 and LG2) to 0.12 (LG8). We observed the total male map length (760.11 cM) was longer compared to the female map (683.71 cM). The largest genomic gap identified was 12.62 cM on LG6.

**Table 2:** Characteristics of the genetic linkage groups of the mapping population from ‘Tom’s special’ and ‘Lacey’.

LG	Number of SNPs	Female length (cM)	Male length (cM)	Sex-avg length (cM)	Average interval (cM)	Maximum gap (cM)
1	1512	97.75	126.40	111.54	0.07	3.38
2	1335	80.34	110.11	95.25	0.07	5.07
3	1315	85.39	130.34	107.59	0.08	2.25
4	953	65.17	88.20	78.10	0.08	3.66
5	766	61.24	67.42	63.77	0.08	2.25
6	731	104.49	87.08	83.02	0.11	12.62
7	570	57.30	50.56	51.13	0.09	1.97
8	547	71.91	60.11	65.48	0.12	4.22
9	494	60.11	39.89	50.46	0.10	10.84
Total	8223	683.71	760.11	706.35	0.09	12.62

### 3.5 QTL analysis

**Figure 3:**
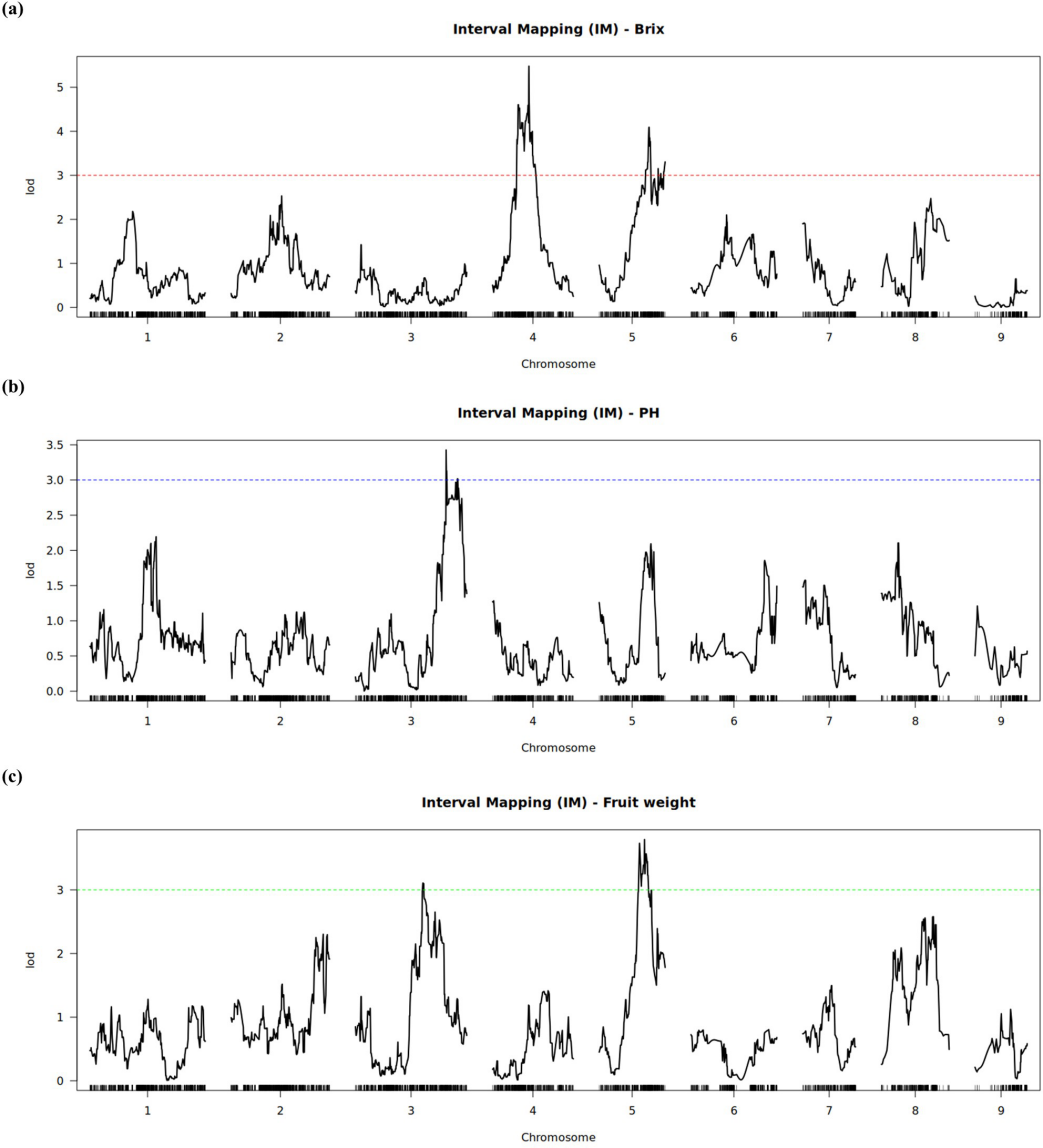
LOD profiles from QTL scans for **(a)** Brix, **(b)** pH, and **(c)** fruit weight across the nine linkage groups.

**Figure 4:**
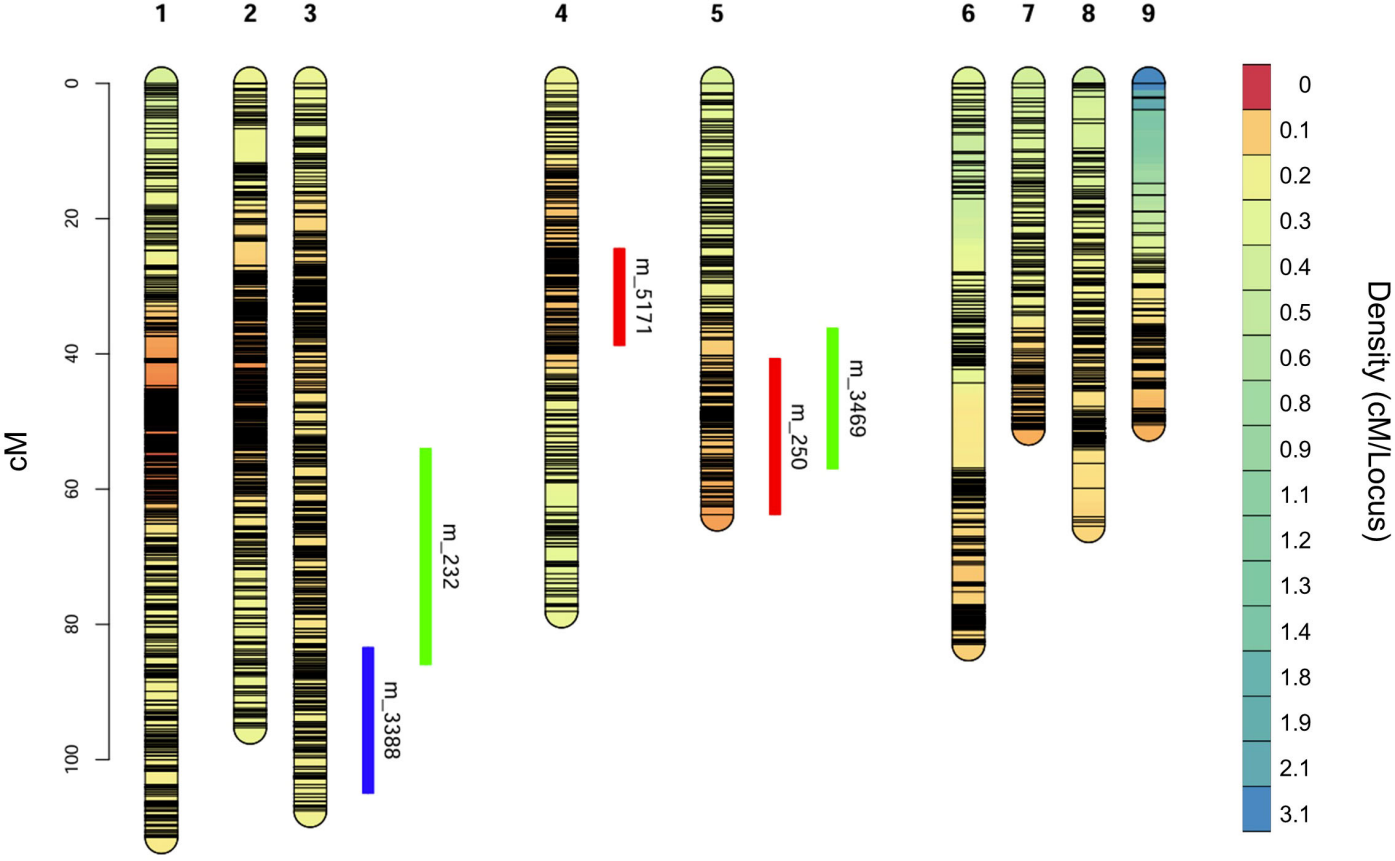
Genetic linkage map (LG1-LG9) of ‘Tom’s special’ (♀) and ‘Lacey’ (♂) progeny population and detected significant quantitative trait loci (QTLs) for Brix (red), pH (blue) and fruit weight (green).

**Figure 5:**
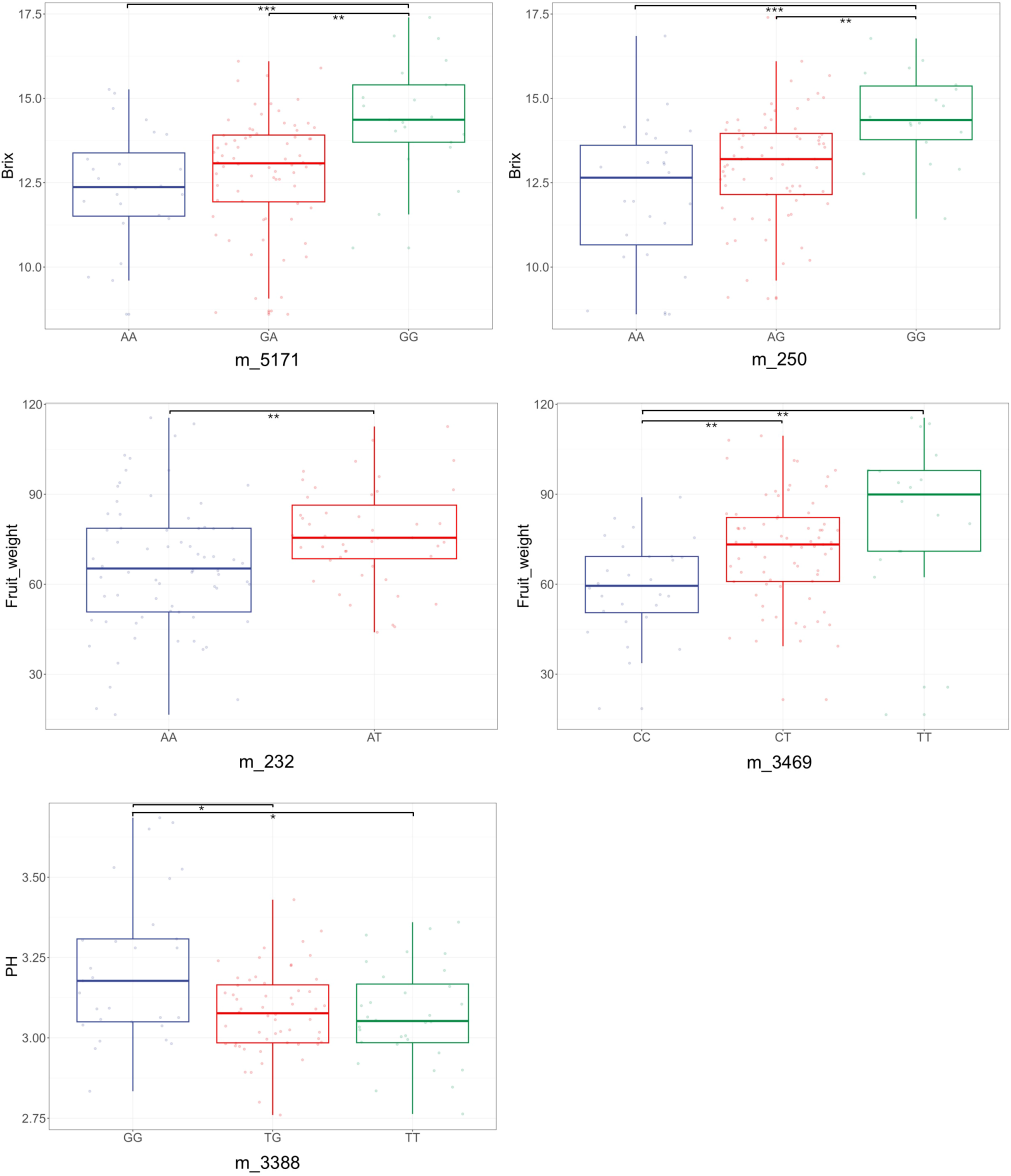
Allelic effects of peak markers associated with three fruit traits. Genotypic states of each marker are shown below each boxplot. Statistically significant differences among genotypes are indicated above the boxplots (*p < 0.05, **p < 0.01, ***p < 0.001).

Based on the developed linkage map and phenotypic data, a total of five QTLs associated with fruit weight and quality were detected using a LOD threshold of 3.0 (**Table 3**). Two significant QTLs associated with Brix were identified (**Figures 3a**, **4**). One major-effect locus, *qBR-4*, was mapped to linkage group 4 and exhibited the highest LOD score (5.48), accounting for 19.40% of the phenotypic variation (**Table 3**). Moreover, the homozygous genotype GG of the peak marker m_5171 showed a positive effect on Brix, with a significant difference between AA and GG (*P* < 0.001) as well as GA and GG (*P* = 0.002) (**Figure 5**). The second QTL of Brix, *qBR-5*, was detected on LG 5, with a peak LOD score of 4.09 associated with marker m_250, explaining 14.87% of the phenotypic variation (**Table 3**). At m_250, the homozygous genotype GG also had a strong positive effect on Brix, differing significantly from AA (*P* < 0.001) and AG (*P* = 0.002) (**Figure 5**).

**Table 3:** Summary of the significant quantitative trait loci (QTLs) detected for Brix (BR), pH content (PH) and fruit weight (FW).

QTL	Trait	LG	P.Marker	Position	Low-Up	Left-Right	LOD	PVE (%)	ADD/*a*	DOM/*d*	*d*/*a*
*qBR-4*	BR	4	m_5171	35.11	24.44-38.77	m_6955-m_3845	5.48	19.40	1.02	-0.59	-0.58
*qBR-5*	BR	5	m_250	48.04	40.74-63.77	m_4190-m_4243	4.09	14.87	1.16	-0.29	-0.25
*qPH-3*	PH	3	m_3388	87.65	83.44-105.00	m_855-m_1411	3.43	12.62	0.07	-0.06	-0.86
*qFW-3*	FW	3	m_232	65.17	54.00-85.96	m_2925-m_4357	3.11	11.51	8.57	4.54	0.53
*qFW-5*	FW	5	m_3469	43.83	36.24-57.00	m_7601-m_7809	3.79	13.86	11.77	0.32	0.03

PVE: the phenotype variance explained (%). P.Marker: peak markers with the highest logarithm of odds (LOD). Position: the genetic position (cM) of P.Marker. Low-Up: the genetic position (cM) of the lower (Left) and upper (Right) boundaries of the 1.5-LOD support interval of each QTL. Left-Right: the closest flanking markers of the 1.5-LOD interval boundaries. ADD/*a*: the additive effect of QTLs. DOM/*d*: the dominance effect of QTLs. *d*/*a*: the degree of dominance of QTLs.

For fruit weight, two QTLs were identified on LG 3 and LG 5 (**Figures 3c**, **4**). The QTL *qFW-3*, with a peak at m_232, explained 11.51% of the phenotypic variation (**Table 3**). The heterozygous genotype AT of m_232 positively affected FW and differed significantly from AA (*P* = 0.005) (**Figure 5**). The second fruit weight QTL, *qFW-5*, was located on LG5 (peak marker m_3469) and explained 13.86% of the phenotypic variance with a significant additive effect (*a* = 11.77) (**Table 3**). However, the CC genotype at m_3469 exhibited a negative effect on fruit weight, with a significantly lower phenotypic mean compared with the CT (*P* = 0.004) and TT (*P* = 0.009) genotypes (**Figure 5**).

Additionally, a single QTL (*qPH-3*), was detected on LG 3 at marker m_3388 (**Figures 3b**, **4**), which contributed 12.62% to the total phenotypic variation of fruit pH (**Table 3**). Analysis of allelic effects at m_3388 further revealed that individuals with the homozygous GG genotype showed significantly higher pH values compared to the heterozygous TG (*P* = 0.014) and homozygous TT (*P* = 0.015) genotypes (**Figure 5**).

The genomic position of these QTLs indicated distinct patterns of potential co-localization. On LG 5, the confidence interval for fruit weight (*qFW-5*; 36.24–57.00 cM) substantially overlapped with that for Brix (*qBR-5*; 40.74–63.77 cM) (**Table 3**; **Figure 4**). The peak markers for these QTLs (m_3469 and m_250) were located approximately 4.2 cM apart. In contrast, on LG 3, the support intervals for fruit weight (*qFW-3*) and pH (*qPH-3*) exhibited only a limited marginal overlap (83.44–85.96 cM) (**Table 3**; **Figure 4**).

### 3.6 Prediction of candidate genes

By aligning the peak marker sequences against the reference genome, four loci were anchored to identify the target genomic regions (**Table 4**). A total of 96 genes were detected within ±100 kb of the associated peak markers (**Supplementary Table 2**), with four key candidate genes revealing functions relevant to fruit development, vacuolar pH regulation, carbohydrate accumulation, and hormone-driven cell expansion (**Table 4**). The analysis revealed that markers m_250 (Brix) and m_3469 (fruit weight) on LG5 (**Figure 4**) were physically mapped to chromosome 6 (chr6) (**Table 4**). Similarly, markers m_3388 (pH) and m_232 (fruit weight) on LG3 (**Figure 4**) were anchored to chromosome 2 (chr2) (**Table 4**). For Brix, the peak marker m_250 was linked to *bHLH68* transcription factor (chr6.899), located approximately 87.8 kb away and implicated in the regulation of carbohydrate accumulation (**Table 4**). Two genomic regions underlying fruit weight were associated with genes involved in developmental and regulatory processes. At a distance of 527 bp, the peak marker m_232 flanked gene chr2.1096, which encodes a *Classical arabinogalactan protein 4-like* (*AGP4*) annotated for fruit growth and development (**Table 4**). Peak marker m_3469 was situated 30.2 kb from *Auxin-responsive protein IAA18* (chr6.641), a key regulator of Auxin signaling and cellular expansion. In addition, the fruit pH-associated marker m_3388 was positioned 90.0 kb from *Sodium/hydrogen exchanger 1* (*NHX1*, chr2.443), which is responsible for cellular pH homeostasis (**Table 4**).

**Table 4:** Key candidate genes of peak markers and their functional annotations in plant species.

Trait	P.Marker	Gene ID	Distance	Gene annotation	Functions	Ref
BR	m_250	chr6.899	87772 bp	Transcription factor bHLH68	Carbohydrate accumulation regulation	(Yu et al., 2021)
PH	m_3388	chr2.443	90027 bp	Sodium/hydrogen exchanger 1	Vacuolar pH regulation	(Bassil et al., 2011)
FW	m_232	chr2.1096	527 bp	Classical arabinogalactan protein 4-like	Fruit growth and development	(Kutyrieva-Nowak et al., 2023b)
FW	m_3469	chr6.641	30207 bp	Auxin-responsive protein IAA18	Auxin signaling and cellular expansion	(Su et al., 2014)

P.Marker: peak markers with the highest logarithm of odds (LOD). Distance: the physical distance between P.Marker and candidate genes (bp).

## 4 Discussion

Passion fruit holds considerable potential for enhancing both productivity and nutritional quality (Thokchom and Mandal, 2017). Elucidating the genetic mechanisms of yield and fruit quality traits is fundamental to advancing the development of enhanced fruit cultivars (Mathiazhagan et al., 2021). Despite the use of various molecular markers in diversity and linkage mapping studies within the *Passiflora* (Cerqueira-Silva et al., 2014), genetic information on QTLs controlling economically significant traits related to yield and fruit quality in *P. edulis* remains limited. In this study, DArT SNP markers were utilized to construct a genetic map derived from a bi-parental population of Australian elite cultivars ‘Tom’s Special’ and ‘Lacey’. A total of 121 progeny from 184 were then phenotyped, and QTL analysis was conducted to identify loci associated with fruit weight and quality. In addition, it is recommended to conduct parentage analysis to exclude selfed individuals due to pollen contamination prior to linkage and QTL analyses and minimize potential bias arising from non-recombinant genotypes, thereby enhancing the reliability of marker–trait associations. The findings of this study enhance our understanding of the molecular mechanisms underlying phenotypic variation, establishing a foundation for future high-resolution fine-mapping and facilitating the development of marker-assisted breeding strategies for passion fruit weight and quality traits.

The improvement of economically important traits relies on a fundamental understanding of the inheritance patterns, the genetic variability available for breeding, and the estimation of genetic parameters (Ribeiro et al., 2019). The limited variation observed in the predicted values of the progeny for Brix, pH, and fruit weight is consistent with the low genomic heritability of three traits (**Table 1**). Brix and fruit weight had higher genomic heritability compared to pH, indicating that additive genetic effects contributed more substantially to the variation in these traits. This finding emphasizes the importance of utilizing these two traits for selection in breeding programs aimed at improving both fruit weight and quality. Previous research on broad-sense heritability (H²) of fruit weight and soluble solids in *P. alata* reported high H² estimates, with genotype-mean heritability values of 0.753 for fruit weight and 0.827 for soluble solids, and individual-plant heritability values of 0.467 and 0.597, respectively (da Silva Pereira et al., 2017). Similarly, de Moraes et al. (2005) reported heritability estimates from a single wide cross in yellow passion fruit as 0.56 for average fruit weight and 0.79 for soluble solids content. Furthermore, a study on sweet passion fruit reported heritability estimates for fruit weight and total soluble solids ranging between 0.45 and 0.89 across varying environmental trials (Chavarría-Perez et al., 2020). The lower heritability observed in our study may be attributed to several factors, including differences in the population studied and variations in the analytical methods used. In this study, we estimated genomic heritability, which represents the proportion of phenotypic variance explained by genetic variation captured through SNPs. Unlike traditional heritability estimates, which rely on pedigree-based relationships, genomic heritability provides marker-based estimates of genetic relationships (Zhu and Zhou, 2020). Lower marker-based heritability derived from genomic best linear unbiased prediction (GBLUP) compared to traditional BLUP has been previously reported in an oilseed crop (Zhao et al., 2021). Fortune et al. (2024) also found that genomic heritability derived from SNPs was lower than broad-sense heritability for most soybean agronomic and seed composition traits. This discrepancy may reflect the presence of missing heritability (Calleja-Rodriguez et al., 2021), indicating the importance of exploring non-additive effects such as dominance and epistasis to better capture the genetic complexity influencing key traits in passion fruit. Since genomic heritability is a crucial factor in evaluating the effectiveness of genomic prediction for traits (Kaler et al., 2022), the genomic parameters estimated in this study will provide valuable insights for the future application of genomic selection in passion fruit breeding, enabling more accurate and efficient selection strategies.

The development of linkage mapping and QTL analysis provides promising opportunities to accelerate genetic improvement and maximize genetic gains in fruit crops (Mathiazhagan et al., 2021). Although the whole genome-wide molecular markers have been widely applied for genetic map construction and exploration of genomic regions of horticultural crops such as peach (Shi et al., 2020), apple (Sun et al., 2015), and pear (Wu et al., 2014), the genomic exploration of passion fruit is still in its early stages due to insufficient genomic resources (Yu et al., 2024). In previous passion fruit genetic mapping studies, Carneiro et al. (2002) and Lopes et al. (2006) utilized RAPD markers and AFLP markers, respectively, to construct genetic maps for the yellow passion fruit, each comprising 9 linkage groups. Subsequently, Oliveira et al. (2008) developed an integrated genetic map for yellow passion fruit with AFLP markers and SSR markers, which included 10 linkage groups. However, these linkage maps were limited by low marker density and insufficient resolution compared with SNP-based platforms. In this study, the use of 8223 SNP markers enabled the construction of a high-density genetic linkage map comprising 9 linkage groups (**Table 2**). As passion fruit is a diploid species with 2n = 18 chromosomes, the identification of nine linkage groups in the present study is consistent with the expected haploid chromosome number. In contrast, the higher number of linkage groups reported in some previous studies likely reflects fragmentation of linkage groups due to differences in mapping population, marker types and density, or linkage parameters (Ferreira et al., 2006; Ronin et al., 2017), rather than true chromosomal differences. The extensive genome-wide distribution, high reproducibility, and scoring accuracy of SNP markers (Maan et al., 2023) substantially enhanced the map resolution, thereby providing a robust framework for the QTL mapping of desirable traits in passion fruit.

Using this genetic linkage map, QTL analysis was conducted to reveal the genetic factors contributing to trait expression of fruit weight and quality in *P. edulis*. We identified five QTLs associated with three fruit traits (Brix, pH, and fruit weight) located on three linkage groups, explaining approximately 11.51% to 19.40% of the phenotypic variance (PVE). Two QTLs associated with Brix were identified on LG4 and LG5, while the QTL linked to pH was mapped to LG3. The QTL for Brix on LG4 can be considered a major QTL, explaining nearly 20% of the phenotypic variance, suggesting a strong genetic contribution to soluble solids content in this population. Additionally, two QTLs associated with fruit weight were located on LG3 and LG5 (**Table 3**). Comparatively, da Silva Pereira et al. (2017) reported four QTLs for soluble solids (PVE of 1.4% to 23.6%) and two QTLs for fruit weight (PVE of 18.2% to 28.4%) located on LG1 and LG4 in *P. alata*. Discrepancies in the number, location, and effect size of QTLs between studies can be attributed to variations in the genetic background of populations, allelic segregation patterns, mapping approaches and marker selection (Collard et al., 2005). Additionally, traits such as Brix and fruit weight are closely linked to source–sink dynamics, assimilate partitioning, and fruit developmental processes that are highly sensitive to environmental factors (Falchi et al., 2020). Therefore, it is crucial to validate QTLs across a range of environments and population backgrounds to ensure their robustness and reliability (Zhaoming et al., 2017).

The identification of overlapping QTL regions for fruit weight and Brix suggests the potential shared genetic control, either through pleiotropy effects of single genes or tight linkage between genes regulating carbohydrate accumulation and fruit growth. Moreover, this genomic co-localization is further supported by the significant positive correlations between Brix and fruit weight for both observed and predicted values (**Figure 2**). Viera et al. (2022) also observed a positive correlation between fruit weight and soluble solids (r = 0.17) for passion fruit germplasm grown in Ecuador. Interestingly, such positive genetic relationships contrast with the findings in tomato, where QTLs for fresh weight and soluble solids content frequently co-localize but exhibit antagonistic effects due to carbon allocation competition between cell structures and sugar metabolism (Prudent et al., 2009). In contrast, the co-localizing QTLs identified in our *P. edulis* underscore the potential presence of favorable alleles associated with increases in both traits. Therefore, it is recommended to utilize this genomic region for simultaneous improvement of both fruit weight and soluble solids content in passion fruit.

The identification of *bHLH68* as a candidate gene linked to Brix in this study (**Table 4**) is supported by functional evidence from apple, where the transcription factor *MdbHLH3* regulates carbohydrate accumulation (Yu et al., 2021). Protein homology analysis further revealed significant structural conservation between passion fruit *bHLH68* and apple *MdbHLH3* (**Supplementary Table 3**). Overexpression of *MdbHLH3* has been shown to enhance fruit sugar levels by modulating photosynthetic capacity and source–sink carbohydrate allocation (Yu et al., 2021). Similarly, our findings suggest that *bHLH68* may play a comparable regulatory role in passion fruit, potentially contributing to the observed variation in soluble solid content. Furthermore, fruit weight was associated with Auxin-responsive protein *IAA18* (**Table 4**). Previous study in tomato provides evidence that Auxin *Sl-IAA17* controls fruit size by modulating cell expansion (Su et al., 2014). Sequence alignment confirmed that passion fruit *IAA18* shares conserved functional domains with the tomato *Sl-IAA17* protein (**Supplementary Table 3**). While our results implicate *IAA18* in the regulation of passion fruit biomass, elucidating its specific mechanisms needs further validation and functional study. The association between fruit weight and *AGP4*, a regulator of cell expansion and differentiation, underscores the importance of cell wall dynamics in fruit size and growth (Kutyrieva-Nowak et al., 2023a, 2023). Interestingly, the detection of *NHX1* for pH highlights the critical influence of intracellular Na^+^/H^+^ antiporters on pH regulation. In Arabidopsis, NHX1 and NHX2 regulate plant growth and reproduction by controlling vacuolar pH and K^+^ homeostasis (Bassil et al., 2011). However, further investigation is required to clarify the specific contribution of *NHX1* to fruit acidity determination. From a physiological perspective, fruit weight and quality are regulated by coordinated carbon and water fluxes involving transpiration, cell division and expansion, seed-derived hormonal signals, and source leaf photosynthetic capacity (Génard et al., 2007). Consequently, integrating physiological and genetic approaches enables a more comprehensive dissection of the genetic basis of these complex traits and their interactions with the environment.

Despite the limitation of single-year data collection in this study, this approach aligns with the standard practice in perennial crop breeding programs to reduce phenotyping costs, minimize maintenance requirements, and accelerate selection cycles (Luby and Shaw, 2001). At early stage, seedlings are typically evaluated in a single location for one year to identify superior genotypes. Selected candidates are subsequently vegetatively propagated for multi-location and multi-year trials. Future studies will focus on validating these markers using grafted clones of the selected genotypes across multiple environments and years to further evaluate genotype-environment (*G* × *E*) interactions.

## 5 Conclusion

This study advanced the understanding of the genetic architecture underlying fruit yield and quality-related traits in Australian passion fruit by constructing a genetic linkage map and identifying genomic regions associated with key traits, including Brix, pH, and fruit weight. Notably, the identification of five novel QTLs and associated candidate genes offer valuable molecular resources for breeding applications. The co-localization of QTLs for fruit weight and Brix, along with their positive genetic correlation, indicates the potential for simultaneous selection of both traits. Moreover, the relatively higher genomic heritability estimates for Brix and fruit weight suggest these traits as promising targets for effective genetic improvement. Future functional validation via tools such as transcriptomics and CRISPR-Cas9 gene editing is essential to confirm the causal roles of these putative candidates. Overall, the findings of this study lay a foundation for the implementation of genomics-assisted selection to accelerate the improvement of fruit weight and quality traits in Australian passion fruit breeding programs.

## Data Availability

The raw data supporting the conclusions of this article will be made available by the authors, without undue reservation.
