# Supplementary material for: Dissecting fruit weight and quality traits in Australian passion fruit through genetic linkage mapping and QTL analysis
Source: Front Plant Sci. 2026 Apr 2;17:1755188. doi: 10.3389/fpls.2026.1755188 (PMC13084201; doi:10.3389/fpls.2026.1755188)
Supplement: Supplementary file 1 [file Table1.docx]

-------------------------------------------------------------------------

# Script Name: QTL_analysis_with_R/qtl.R

# Purpose: Statistical analysis of marker-trait association

# Author: Xinhang Sun

# Date: 21/01/2026

# Paper: Dissecting fruit weight adn quality traits in Australian passion fruit through genetic linkage mapping and QTL analysis

# Data required: "QTL_input.csv"

# -------------------------------------------------------------------------

library(qtl)

# # Load library

#

# Import data

phenogeno <- read.cross(format = "csv",

file = "QTL_input.csv",

genotypes = NULL,

crosstype = "4way")

# Explore data

summary(phenogeno)

plot(phenogeno)

# Fix map order jitter if needed

qtl.data <- jittermap(phenogeno, amount = 1e-6)

# Calculate genotype probabilities

qtl.d <- calc.genoprob(

qtl.data,

step = 1, # 1 cM spacing

off.end = 0,

error.prob = 0.001,

stepwidth = "fixed",

map.function = "kosambi"

)

# Perform genome-wide QTL scan (interval mapping) for Brix

scan.res <- scanone(

qtl.data,

pheno.col = 1)

# Plot LOD profile

plot(scan.res, main = "Interval Mapping (IM) - Brix")

# Permutation test for significance thresholds

perm.res <- scanone(

qtl.d,

pheno.col = 1, n.perm = 1000)

# Determine 5% genome-wide threshold

lod.threshold <- summary(perm.res, alpha = 0.1)

lod.threshold

# plot(scan.res, main = "Interval Mapping (IM) LOD Profile")

abline(h = lod.threshold, col = "red", lty = 2)

# Summarize significant QTLs

sig.qtl <- summary(scan.res, threshold = lod.threshold, format = "tabByChr")

sig.qtl

# Estimate confidence interval for first significant QTL

ci <- lodint(scan.res, chr = 5, drop = 1.5)

print(ci)

ci <- lodint(scan.res, chr = 4, drop = 1.5)

print(ci)

# Create QTL model from detected peaks

qtl.model <- makeqtl(

qtl.d,

chr = 5,

pos = 48.32030,

what = "prob")

# Fit model and estimate effects

fit <- fitqtl(

qtl.d, # use the same cross object

qtl = qtl.model,

pheno.col = 1,

method = "hk",get.ests = T)

summary(fit)

-------------------------------------------------------------------------

# Script Name: Linkage_map_visualization_linkagemapview.R

# Purpose: To visualize linkage map with or without QTLs highlighted

# Author: Xinhang Sun

# Date: 21/01/2026

# Paper: Dissecting fruit weight adn quality traits in Australian passion fruit through genetic linkage mapping and QTL analysis

# Data required: "pf_map.txt","qtl_data.csv"

# -------------------------------------------------------------------------

# Install and load the package

install.packages("LinkageMapView")

library(LinkageMapView)

library(readxl)

# Read your linkage map file (adjust the filename!)

map_data <- read.table("pf_map.txt",header=T)

# Rename columns if needed

colnames(map_data) <- c("Group", "Position", "Locus")

# Sort by linkage group and position

summary(map_data)

str(map_data)

# Get all linkage groups

lg_to_plot <- sort(unique(map_data$Group)) # expects 1–9

# Compute axis range (0–max cM across all LGs)

maxpos <- floor(max(map_data$Position))

at.axis <- seq(0, maxpos)

axlab <- vector()

for (lab in 0:maxpos) {

if (!lab %% 10) {

axlab <- c(axlab, lab)

}

else {

axlab <- c(axlab, NA)

}

}

# Draw all linkage groups in one map

lmv.linkage.plot(

map_data,

outfile,

mapthese = lg_to_plot, # all LGs

denmap = TRUE, # density visualization

cex.axis = 0.8, # axis text size

at.axis = at.axis,

labels.axis = axlab,

)

graphics.off()

par(mfrow = c(1,1))

outfile <- file.path(tempdir(), "All_Linkage_Groups.pdf")

lmv.linkage.plot(

map_data,

outfile = "pf_map.pdf",

mapthese = sort(unique(map_data$Group)),

denmap = TRUE,

pdf.width = 9,

pdf.height = 10,# show on screen, no PDF

cex.axis = 0.8

)

# for map with QTLs

qtldf <- read.csv("qtl_data.csv")

qtldf$chr = as.character(qtldf$chr)

qtldf$qtl = as.character(qtldf$qtl)

qtldf$so = as.numeric(qtldf$so)

qtldf$si = as.numeric(qtldf$si)

qtldf$ei = as.numeric(qtldf$ei)

qtldf$eo = as.numeric(qtldf$eo)

qtldf$col = as.character(qtldf$col)

# Set output file

outfile <- "pf_map_with_QTL.pdf"

# Plot linkage map with QTLs highlighted

lmv.linkage.plot(

map = map_data,

outfile = "pf_map_with_QTL.png",

mapthese = sort(unique(map_data$Group)),

denmap = TRUE,

qtldf=qtldf,

cex.axis = 0.8,

pdf.width = 9,

pdf.height = 10

)
