# Supplementary material for: Dissecting fruit weight and quality traits in Australian passion fruit through genetic linkage mapping and QTL analysis
Source: Front Plant Sci. 2026 Apr 2;17:1755188. doi: 10.3389/fpls.2026.1755188 (PMC13084201; doi:10.3389/fpls.2026.1755188)
Supplement: Supplementary file 2 [file Table2.docx]

Supplementary Material

# Supplementary Figures and Tables

**Supplementary Table 1.** List of selfed individuals from the results of parentage analysis.

| **Offspring** | **Parent1** | **Parent2** | **Type** | **GD** | **p.value** |
| --- | --- | --- | --- | --- | --- |
| TxL-156 | Tom’s Special | Tom’s Special.1 | self | 0.016 | 0.000 |
| TxL-180 | Tom’s Special | Tom’s Special.1 | self | 0.038 | 0.000 |
| TxL-27 | Tom’s Special | Tom’s Special.1 | self | 0.039 | 0.000 |
| TxL-194 | Tom’s Special | Tom’s Special.1 | self | 0.040 | 0.000 |
| TxL-57 | Tom’s Special | Tom’s Special.1 | self | 0.047 | 0.000 |
| TxL-173 | Tom’s Special | Tom’s Special.1 | self | 0.048 | 0.000 |

**Supplementary Table 2.**  Functional annotation of all candidate genes identified within the ±100 kb intervals flanking the four anchored peak markers.

| **Trait** | **P.Marker** | **Gene ID** | **Distance (bp)** | **Gene annotation** |
| --- | --- | --- | --- | --- |
| **BR** | **m_250** | **chr6.899** | **87772** | **Transcription factor bHLH68** |
|  |  | chr6.900 | 86592 | Uncharacterized protein |
|  |  | chr6.901 | 0 | Bark storage protein A |
|  |  | chr6.902 | 1969 | U-box domain-containing protein 11 |
|  |  | chr6.903 | 9814 | Peroxiredoxin-2 |
|  |  | chr6.904 | 13403 | Peroxiredoxin-2 |
|  |  | chr6.905 | 16282 | Rac-like GTP-binding protein ARAC7 |
|  |  | chr6.906 | 30258 | Cyclin-dependent kinase F-1 |
|  |  | chr6.907 | 33072 | 18S rRNA (guanine-N(7))-methyltransferase RID2 |
| **PH** | **m_3388** | chr2.405 | 98059 | Probable E3 ubiquitin-protein ligase BAH1-like 1 |
|  |  | chr2.406 | 95208 | Probable E3 ubiquitin-protein ligase BAH1-like 1 |
|  |  | chr2.407 | 93688 | Uncharacterized protein |
|  |  | chr2.408 | 86641 | ABC transporter C family member 14 |
|  |  | chr2.409 | 77021 | Uncharacterized protein |
|  |  | chr2.410 | 73034 | Uncharacterized protein |
|  |  | chr2.411 | 70584 | Probable U6 snRNA-associated Sm-like protein LSm4 |
|  |  | chr2.412 | 58467 | Outer mitochondrial transmembrane helix translocase |
|  |  | chr2.413 | 54696 | Alkylated DNA repair protein alkB homolog 8 |
|  |  | chr2.414 | 52698 | Rapid alkalinization factor |
|  |  | chr2.415 | 43358 | Probable indole-3-acetic acid-amido synthetase GH3.11 |
|  |  | chr2.416 | 39918 | Ferritin-like catalase Nec2 |
|  |  | chr2.417 | 36797 | Dephospho-CoA kinase |
|  |  | chr2.418 | 33368 | Glutathione S-transferase |
|  |  | chr2.419 | 29958 | Glutathione S-transferase PARB |
|  |  | chr2.420 | 28351 | Glutathione S-transferase |
|  |  | chr2.421 | 26820 | Glutathione S-transferase |
|  |  | chr2.422 | 24993 | Glutathione S-transferase |
|  |  | chr2.423 | 12435 | Glutathione S-transferase F13 |
|  |  | chr2.424 | 7404 | Autophagy-related protein 18a |
|  |  | chr2.425 | 1409 | Uncharacterized protein |
|  |  | chr2.426 | 901 | Nuclear transcription factor Y subunit B-5 |
|  |  | chr2.427 | 5429 | Uncharacterized protein |
|  |  | chr2.428 | 9092 | Protein indeterminate-domain 7 |
|  |  | chr2.429 | 15508 | Cysteine-tryptophan domain-containing zinc finger protein 7 |
|  |  | chr2.430 | 27568 | Peroxisomal ATPase PEX6 |
|  |  | chr2.431 | 37265 | Zinc finger CCCH domain-containing protein 32 |
|  |  | chr2.432 | 43478 | POZ domain-containing protein At1g03010 |
|  |  | chr2.433 | 50788 | Monothiol glutaredoxin-S1 |
|  |  | chr2.434 | 54901 | Monothiol glutaredoxin-S1 |
|  |  | chr2.435 | 58740 | Monothiol glutaredoxin-S6 |
|  |  | chr2.436 | 61224 | OVARIAN TUMOR DOMAIN-containing deubiquitinating enzyme 5 |
|  |  | chr2.437 | 65838 | Glutaredoxin-C11 |
|  |  | chr2.438 | 69195 | Glutaredoxin-C13 |
|  |  | chr2.439 | 71730 | Uncharacterized protein |
|  |  | chr2.440 | 75689 | Protein TRANSPORT INHIBITOR RESPONSE 1 |
|  |  | chr2.441 | 84182 | Uncharacterized protein |
|  |  | chr2.442 | 85818 | Tubby-like F-box protein 3 |
|  |  | **chr2.443** | **90027** | **Sodium/hydrogen exchanger 1** |
|  |  | chr2.444 | 95579 | Classical arabinogalactan protein 26 |
| **FW** | **m_232** | chr2.1086 | 98383 | Bet1-like protein At4g14600 |
|  |  | chr2.1087 | 96061 | Uncharacterized protein |
|  |  | chr2.1088 | 91916 | Purine permease 3 |
|  |  | chr2.1089 | 41706 | Protein TRACHEARY ELEMENT DIFFERENTIATION-RELATED 7A |
|  |  | chr2.1090 | 29470 | Protein DETOXIFICATION 27 |
|  |  | chr2.1091 | 26766 | RING-H2 finger protein ATL11 |
|  |  | chr2.1092 | 19621 | Transcription factor bHLH94 |
|  |  | chr2.1093 | 12747 | RING-H2 finger protein ATL54 |
|  |  | chr2.1094 | 5128 | Uncharacterized protein |
|  |  | chr2.1095 | 1858 | RNA-binding protein CP33, chloroplastic |
|  |  | **chr2.1096** | **527** | **Classical arabinogalactan protein 4-like** |
|  |  | chr2.1097 | 5837 | Blue copper protein |
|  |  | chr2.1098 | 11512 | Protein FAM91A1 |
|  |  | chr2.1099 | 23318 | Homeobox-leucine zipper protein ATHB-5 |
|  |  | chr2.1100 | 26936 | 39S ribosomal protein L41-A, mitochondrial |
|  |  | chr2.1101 | 29883 | Uncharacterized protein |
|  |  | chr2.1102 | 33575 | O-fucosyltransferase 7 |
|  |  | chr2.1103 | 38630 | Thylakoid lumenal 29 kDa protein, chloroplastic |
|  |  | chr2.1104 | 44085 | Serine--tRNA ligase, chloroplastic/mitochondrial |
|  |  | chr2.1105 | 51056 | 14-3-3-like protein |
|  |  | chr2.1106 | 56238 | Serine/threonine-protein kinase SAPK2 |
|  |  | chr2.1107 | 59860 | Phospho-2-dehydro-3-deoxyheptonate aldolase 1, chloroplastic |
|  |  | chr2.1108 | 68824 | F-box protein FBW2 |
|  |  | chr2.1109 | 75695 | Serine/threonine-protein phosphatase 2A activator |
|  |  | chr2.1110 | 79764 | Protein EXORDIUM-like 2 |
|  |  | chr2.1111 | 83876 | Probable protein phosphatase 2C 10 |
|  |  | chr2.1112 | 90577 | Monoacylglycerol lipase ABHD6 |
|  |  | chr2.1113 | 93155 | 14-3-3 protein 7 |
|  |  | chr2.1114 | 98184 | Melanoma-associated antigen 1 |
| **FW** | **m_3469** | chr6.636 | 99478 | Uncharacterized protein |
|  |  | chr6.637 | 93688 | Caffeoylshikimate esterase |
|  |  | chr6.638 | 91050 | Uncharacterized protein |
|  |  | chr6.639 | 46868 | Casein kinase 1-like protein HD16 |
|  |  | chr6.640 | 37526 | Retrovirus-related Pol polyprotein from transposon TNT 1-94 |
|  |  | **chr6.641** | **30207** | **Auxin-responsive protein IAA18** |
|  |  | chr6.642 | 23580 | Receptor-like protein kinase 5 |
|  |  | chr6.643 | 18369 | Nuclear poly(A) polymerase 4 |
|  |  | chr6.644 | 15481 | Profilin (Fragment) |
|  |  | chr6.645 | 9155 | ATP-dependent 6-phosphofructokinase 3 |
|  |  | chr6.646 | 0 | Calcineurin-binding protein 1 |
|  |  | chr6.647 | 2072 | Glutamyl-tRNA(Gln) amidotransferase subunit C, chloroplastic/mitochondrial |
|  |  | chr6.648 | 3885 | RNA polymerase sigma factor sigC |
|  |  | chr6.649 | 23289 | Zinc finger CCCH domain-containing protein 20 |
|  |  | chr6.650 | 30208 | Transcription factor RAX3 |
|  |  | chr6.651 | 34476 | Clathrin interactor EPSIN 1 |
|  |  | chr6.652 | 42449 | Glycosyltransferase BC10 |
|  |  | chr6.653 | 49011 | Uncharacterized protein |

**Supplementary Table 3.** Homology-based validation of passion fruit candidate genes.

| **Candidate** | **Model Ortholog** | **Trait** | **Identity (%)** | **Positives (%)** | **E-value** |
| --- | --- | --- | --- | --- | --- |
| *IAA18* | *Sl-IAA17* Tomato | Fruit Weight | 36.0 | 51.0 | 1 × 10^-29^ |
| *bHLH68* | *MdbHLH3* Apple | Brix | 34.0 | 52.0 | 2 × 10^-7^ |


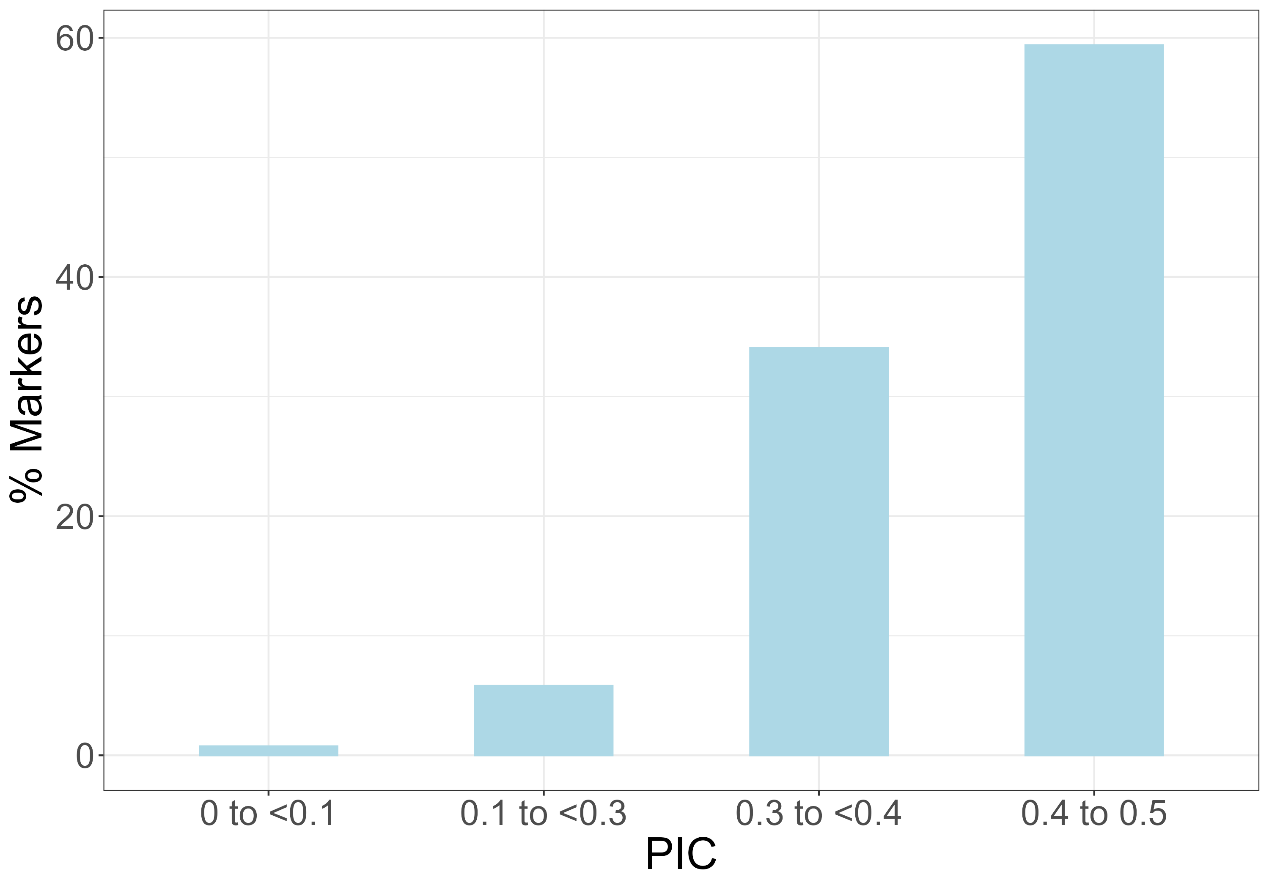


**Supplementary Figure 1.** Distribution of PIC values for 11506 SNP markers used in the study.
